# Supplementary material for: Identification of Freezing-Responsive microRNAs and Their Targets in Chinese Jujube by Small RNA and Degradome Sequencing
Source: Int J Mol Sci. 2026 Sep 4;27(17):7912. doi: 10.3390/ijms27177912 (PMC13566890; doi:10.3390/ijms27177912)
Supplement: Supplementary file 1 [file ijms-27-07912-s001.zip › Supplementary Material S2/novel_miR_147.pdf]

Provisional ID : novel_miR_147
Score total : 545.5
Score for star read(s) : 3.9
Score for read counts : 537.5
Score for mfe : 2.5
Score for randfold : 1.6
Score for cons. seed :
Total read count : 1066
Mature read count : 692
Loop read count : 1
Star read count : 373

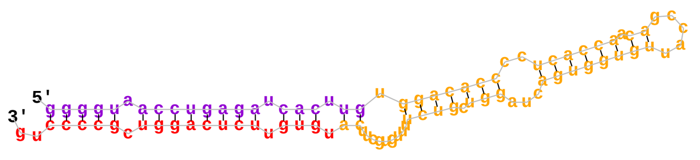

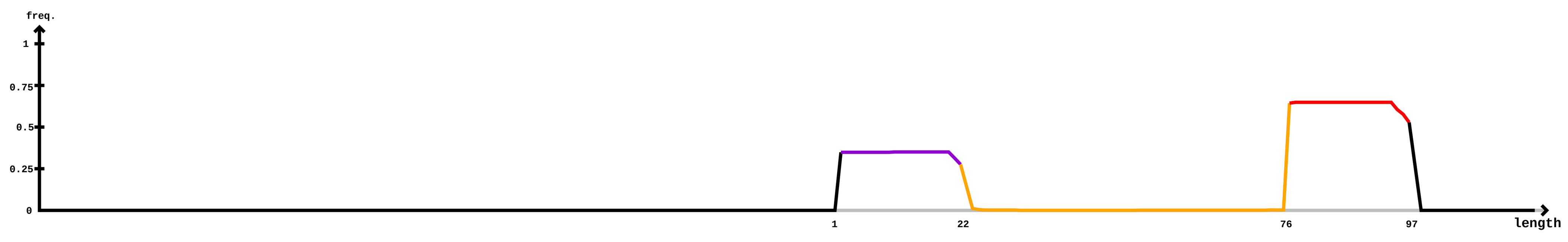

Star Mature

```
5'- auuaucauguacuaugguguaguacaaguuuagucaaguacucaaucaagcaggagcuguuguuauauaacuugguauaaacugagugagcaucacaagcacagccauguuaagagguaaaggagucccacagggguaaccugagaucacuuguggacaccccucaccaacagccauuguggugacuaggucgucuuuugugcuucauguguucucaggucgccccugcagggcuucccuuuaccacaaa -3'  obs
    auuaucauguacuaugguguaguacaaguuuagucaaguacucaaucaagcaggagcuguuguuauauaacuugguauaaacugagugagcaucacaagcacagccauguuaagagguaaaggagucccacagggguaaccugagaucacuuguggacaccccucaccaacagccauuguggugacuaggucgucuuuugugcuucauguguucucaggucgccccugcagggcuucccuuuaccacaaa      exp
    .......(((.((.((((((..(((.((((((.((((((........(((((...)))))........))))))..))))))..))).))))))..)).)))..............((((((((((((..(((((((.((((((((.(((.((.(((((((..((((((.((.....))))))))...))).))))........)).))).))))))))).)))))))..)))))..)))))))).....  reads  mm  sample
    ....................................................................................................................................gggguaaccugagaucacu...................................................................................................  6  0  JT-3
    ....................................................................................................................................gggguaaccugagaucacu...................................................................................................  1  0  JCK-2
    ....................................................................................................................................gggguaaccugagaucacu...................................................................................................  7  0  LCK-3
    ....................................................................................................................................gggguaaccugagaucacu...................................................................................................  4  0  LT-1
    ....................................................................................................................................gggguaaccugagaucacu...................................................................................................  3  0  JCK-1
    ....................................................................................................................................gggguaaccugagNucacu...................................................................................................  1  1  JT-2
    ....................................................................................................................................gggguaaccugagaucacu...................................................................................................  2  0  JCK-3
    ....................................................................................................................................gggguaaccugagaucacu...................................................................................................  7  0  LT-2
    ....................................................................................................................................gggguaaccugagaucacu...................................................................................................  1  0  JT-2
    ....................................................................................................................................gggguaaccugagaucacu...................................................................................................  7  0  LT-3
    ....................................................................................................................................gggguaaccugagaucacuu..................................................................................................  2  0  LT-2
    ....................................................................................................................................gggguaaccugagaucacuu..................................................................................................  1  0  LT-3
    ....................................................................................................................................gggguaaccugagaucacuu..................................................................................................  6  0  JCK-3
    ....................................................................................................................................gggguaaccugagaucacuu..................................................................................................  12  0  JCK-1
    ....................................................................................................................................gggguaaccugagaucacuu..................................................................................................  3  0  LT-1
    ....................................................................................................................................gggguaaccugagaucacuu..................................................................................................  17  0  JCK-2
    ....................................................................................................................................gggguaaccugagaucacuug.................................................................................................  96  0  JCK-2
    ....................................................................................................................................gggguaaccugagaucacuug.................................................................................................  11  0  LT-3
    ....................................................................................................................................gggguaaccugagaucacuug.................................................................................................  4  0  LT-2
    ....................................................................................................................................gNgguaaccugagaucacuug.................................................................................................  1  1  JCK-1
    ....................................................................................................................................gggguaaccugagaucacuug.................................................................................................  41  0  JCK-1
    ....................................................................................................................................gggguaaccugagaucacuuU.................................................................................................  4  1  JCK-1
    ....................................................................................................................................gggguaaccugagaucacuuU.................................................................................................  5  1  JCK-2
    ....................................................................................................................................gggguaaccugagaucacuug.................................................................................................  15  0  LCK-3
    ....................................................................................................................................gggguaaccugagaucacuuU.................................................................................................  2  1  JCK-3
    ....................................................................................................................................gggguaacAugagaucacuug.................................................................................................  1  1  JCK-2
    ....................................................................................................................................gggguaaccugagaucacuug.................................................................................................  3  0  JT-2
    ....................................................................................................................................gggguaaccugagaucacuug.................................................................................................  58  0  JCK-3
    ....................................................................................................................................gggguaaccugagaucacuug.................................................................................................  9  0  JT-3
    ....................................................................................................................................gggguaaccugagaucacuugC................................................................................................  1  1  JCK-3
    ....................................................................................................................................gggguaaccugagaucacuugA................................................................................................  4  1  LCK-3
    ....................................................................................................................................gggguaaccugagaucacuugA................................................................................................  6  1  LT-2
    ....................................................................................................................................gggguaaccugagaucacuuUu................................................................................................  1  1  LT-2
```

```
                                                                                                                                    Star                                                                            Mature
auuaucauguacuaugguguaguacaaguuuagucaaguacucaaucaagcaggagcuguuguuauauaacuugguauaaacugagugagcaucacaagcacagccauguuaagagguaaaggagucccacagggguaaccugagaucacuuguggacaccccucaccaacagccauugguggugacuaggucgucuuuugugcuucauguguucucaggucgccccugcagggcuucccuuuaccacaaa
....................................................................................................................................gggguaaccugagaucacuugA.................................................................................................  7    1    JCK-3
....................................................................................................................................gggguaaccugagaucacuugA.................................................................................................  1    1    LT-3
....................................................................................................................................gggguaaccugagaucacuugA.................................................................................................  1    1    JCK-1
....................................................................................................................................gggguaaccugagaucacuugu.................................................................................................  3    0    JCK-2
....................................................................................................................................gggguaaccugagaucacuugA.................................................................................................  9    1    JCK-2
....................................................................................................................................gggguaaccugagaucacuugug................................................................................................  7    0    JCK-2
....................................................................................................................................gggguaaccugagaucacuugugg...............................................................................................  3    0    JCK-3
.............................................................................................................................................ugagaucacuuguggacaccc.........................................................................................  2    0    LT-2
.......................................................................................................................................................................................ugacuaggucgucuuuugugcuu.............................................  1    0    LT-1
.............................................................................................................................................................................................................ucauguguucucaggucgccccug......................  1    0    JCK-3
.............................................................................................................................................................................................................ucauguguucucaggucgccccug......................  1    0    JCK-1
...............................................................................................................................................................................................................auguguucucaggucgccccu.......................  1    0    LT-1
................................................................................................................................................................................................................uguguucucaggucgccc.........................  7    0    JCK-2
................................................................................................................................................................................................................uguguucucaggucgccc.........................  2    0    LCK-3
................................................................................................................................................................................................................uguguucucaggucgccc.........................  6    0    LT-3
................................................................................................................................................................................................................uguguucucaggucgccc.........................  2    0    LT-1
................................................................................................................................................................................................................uguguucucaggucgccc.........................  6    0    LT-2
................................................................................................................................................................................................................uguguucucaggucgccc.........................  2    0    JCK-3
................................................................................................................................................................................................................uguguucucaggucgccc.........................  18   0    JT-1
................................................................................................................................................................................................................uguguucucaggucgccc.........................  3    0    JCK-1
................................................................................................................................................................................................................uguguucucaggucgcccc........................  11   0    LT-3
................................................................................................................................................................................................................uguguucucaggucgcccc........................  2    0    JCK-1
................................................................................................................................................................................................................uguguucucaggucgcccc........................  5    0    LT-2
................................................................................................................................................................................................................uguguucucaggucgcccc........................  3    0    JT-1
................................................................................................................................................................................................................uguguucucaggucgcccc........................  3    0    LCK-3
................................................................................................................................................................................................................uguguucucaggucgcccc........................  6    0    LT-1
................................................................................................................................................................................................................uguguucucaggucgccccu.......................  1    0    JT-3
................................................................................................................................................................................................................uguguucucaggucgccccu.......................  12   0    LT-2
................................................................................................................................................................................................................uguguucucaggucgccccu.......................  1    0    JT-2
................................................................................................................................................................................................................uguguucucaggucgccccu.......................  7    0    JCK-1
................................................................................................................................................................................................................uguguucucaggucgccccu.......................  6    0    JCK-3
................................................................................................................................................................................................................uguguucucaggucgccccA.......................  1    1    JT-1
................................................................................................................................................................................................................uguguucucaggucgccccu.......................  2    0    LCK-3
................................................................................................................................................................................................................uguguucucaggucgccccu.......................  8    0    LT-3
................................................................................................................................................................................................................uguguucucaggucgccccu.......................  8    0    JCK-2
................................................................................................................................................................................................................uguguucucaggucgccccu.......................  4    0    LT-1
................................................................................................................................................................................................................uguguucucaggucgccccuA......................  1    1    JCK-2
................................................................................................................................................................................................................uguguucucaggucgccccug......................  76   0    LCK-1
................................................................................................................................................................................................................uguguucucaggucgccccAg......................  3    1    JCK-2
................................................................................................................................................................................................................uguguucucaggucgccccug......................  2    0    JT-2
................................................................................................................................................................................................................uguguucucaggucgccccuU......................  1    1    JCK-1
................................................................................................................................................................................................................uguguucucaggucgccccuC......................  1    1    JCK-1
................................................................................................................................................................................................................uguguucucaggucgccccuC......................  1    1    LT-3
................................................................................................................................................................................................................Cguguucucaggucgccccug......................  1    1    JCK-1
................................................................................................................................................................................................................uguguucucaggucgccccug......................  103  0    LCK-2
................................................................................................................................................................................................................uguguucucaggucgccccuU......................  7    1    JCK-3
................................................................................................................................................................................................................uguguucucaggucgccccug......................  74   0    JCK-3
................................................................................................................................................................................................................uguguucucaggucgccccuU......................  11   1    JCK-2
................................................................................................................................................................................................................uguguucucaggucgccccAg......................  1    1    JCK-1
................................................................................................................................................................................................................uguguucucagguNgccccug......................  1    1    LCK-1
................................................................................................................................................................................................................uguguucucaggucgccccug......................  9    0    LT-1
................................................................................................................................................................................................................uguguucucaggucgccccug......................  7    0    JT-3
................................................................................................................................................................................................................uNuguucucaggucgccccug......................  2    1    JCK-2
................................................................................................................................................................................................................uguguucucaggucgccccug......................  13   0    LT-3
................................................................................................................................................................................................................uNuguucucaggucgccccug......................  1    1    LCK-1
................................................................................................................................................................................................................uguguucucaggucgccccug......................  56   0    JT-1
................................................................................................................................................................................................................uguguucucaggucgccccug......................  99   0    JCK-2
................................................................................................................................................................................................................uguguucucaggucgccccuA......................  5    1    JCK-3
................................................................................................................................................................................................................uguguucucaggucgcAccug......................  1    1    JCK-2
................................................................................................................................................................................................................uguguucucaggucgccccug......................  70   0    JCK-1
................................................................................................................................................................................................................uguguucucaggucgccccug......................  4    0    LT-2
................................................................................................................................................................................................................uguguucucaggucgccccug......................  7    0    LCK-3
................................................................................................................................................................................................................uguguucucaggucgcccAug......................  1    1    LCK-2
................................................................................................................................................................................................................uguguucucaggucgAcccug......................  1    1    JCK-2
................................................................................................................................................................................................................uguguucucaggucgccccuA......................  1    1    JCK-1
.................................................................................................................................................................................................................guguucucaggucgcccc........................  1    0    LT-3
.................................................................................................................................................................................................................guguucucaggucgccccu.......................  1    0    JCK-2
.................................................................................................................................................................................................................guguucucaggucgccccug......................  2    0    JCK-2
.................................................................................................................................................................................................................guguucucaggucgccccug......................  1    0    JCK-1
```
